# Retraction Note: Vitamin D regulates insulin and ameliorates apoptosis and oxidative stress in pancreatic tissues of rats with streptozotocin-induced diabetes

**DOI:** 10.1007/s11356-026-38030-1

**Published:** 2026-07-03

**Authors:** Fatima El Zahra M. Fathi, Kadry M. Sadek, Asmaa F. Khafaga, Abdel Wahab Alsenosy, Hanan A. Ghoniem, Sahar Fayez, Mohamed F. Zeweil

**Affiliations:** 1 Department of Biochemistry, Faculty of Veterinary Medicine, Damanhur University, Damanhour, 22516 Egypt; 2 Department of Pathology, Faculty of Veterinary Medicine, Alexandria University, Edfina, 22758 Egypt; 3 Department of Physiology, Faculty of Veterinary Medicine, Damanhur University, Damanhour, 22516 Egypt; 4 Department of Histology and Cytology, Faculty of Veterinary Medicine, Damanhur University, Damanhour, 22516 Egypt

**Retraction Note: Environmental Science and Pollution Research (2022) 29:90219–90229**

10.1007/s11356-022-22064-2

The Editor-in-Chief and the Publisher have retracted this article as the editorial and peer review processes appear to have been compromised. Based on this, the Editor-in-Chief no longer has confidence in the results and conclusions of the article.

Authors Asmaa F. Khafaga, Kadry M. Sadek and Mohamed F. Zeweil disagree with this retraction. Authors Abdel Wahab Al senosy, Hanan A. Ghoniem and Sahar Fayez did not respond to correspondence from the Publisher about this retraction. The Publisher could not obtain a current email address for author Fatima El Zahra M. Fathi.

